# Cardiac and autonomic function in patients with Wilson’s disease

**DOI:** 10.1186/s13023-019-1007-7

**Published:** 2019-01-28

**Authors:** Silvio Quick, Ulrike Reuner, Marie Weidauer, Charlotte Hempel, Felix Martin Heidrich, Christoph Mues, Krunoslav Michael Sveric, Karim Ibrahim, Heinz Reichmann, Axel Linke, Uwe Speiser

**Affiliations:** 1 Department for Internal Medicine and Cardiology, Herzzentrum Dresden, Technische Universität Dresden, Fetscherstr. 76, 01307 Dresden, Germany; 2 Department of Neurology, University Clinic Dresden, Technische Universität Dresden, Fetscherstr. 74, 01307 Dresden, Germany

**Keywords:** Wilson’s disease, Autonomic dysfunction, Cardiac involvement, Heart failure, Echocardiography, Unified Wilson’s disease rating scale

## Abstract

**Background:**

The clinical effect of copper accumulation on the heart of patients suffering from Wilson’s disease (WD) is not completely understood. We aimed to determine if patients with WD show signs of cardiac involvement, structural heart disease or autonomic dysfunction. In this prospective trial, we studied 61 patients (mean age 44.3 ± 15.2 years, 51% males) with WD and compared them to 61 age- and gender-matched healthy controls. All subjects underwent clinical examination, blood tests, echocardiography and 24 h electrocardiographic (ECG) recording.

**Results:**

Left- and right ventricular systolic function did not differ significantly between WD patients and controls. However, 5 of the 61 patients had a reduced left ventricular ejection fraction (LVEF). Furthermore, diastolic dysfunction was more prevalent in WD patients (9 of 61 vs. 0 of 61, *p* = 0.001).

The severity of WD based on the Unified Wilson’s Disease Rating Scale was significantly correlated to NT-pro BNP (r = 0.34, *P* = 0.013). Patients with an exacerbation of WD in medical history had higher troponin levels compared to those without (11.3 ± 4.7 vs 4.6 ± 1.2).

The autonomic function assessed by triangular index (TI) and SDNN-index was significantly reduced in WD patients compared to controls in most in almost every age category (*p*-value TI and SDNN: age 20–29, *p* < 0.001 and 0.05; age 30–39, *p* < 0.01 and not significant (ns); age 40–49, *p* < 0,01 and 0.001; age 50–59, *p* = ns and < 0.001, age 60–70, *p* < 0.05 and ns).

**Conclusion:**

Our data demonstrate that cardiac involvement and autonomic dysfunction in WD is possible, however the underlying cause is still not known. We suggest that patients with signs and symptoms of structural heart disease should be examined by a cardiologist in addition to the interdisciplinary treatment team of WD.

**Electronic supplementary material:**

The online version of this article (10.1186/s13023-019-1007-7) contains supplementary material, which is available to authorized users.

## Background

Wilson’s disease (WD) is an inherited autosomal recessive disorder resulting from abnormal copper metabolism. It was first described by Kinnier Wilson in 1912. WD leads to reduced copper excretion and causes an excessive deposition of the copper into different organs, including the liver, the central nervous system, the cornea, the kidney and joints [1]. WD is rare,with an averaged incidence in the world of 1:30.000 inhabitants that may reaches significantly higher peaks in some isolated geographic areas [2]. However, relatively little is known about the effects of copper accumulation on the heart. Kaduk et al. described a case of cardiomyopathy in WD. Myocardial copper accumulation was revealed by electron microscope [3]. Furthermore, Factor et al. could demonstrate increased copper values in the myocardium in an autopsy study in 5 of 9 patients suffering from WD [4]**.**

It was recently reported that WD patients have a significantly elevated risk of both atrial fibrillation and heart failure [5], but signs of structural heart disease have not yet been found. Some smaller WD studies, focusing on electrocardiographic changes and standard echocardiographic parameters, showed a mild, non-pathological increase in the thickness of the interventricular septum and the posterior wall [6]. For that reason, cardiac involvement in WD has generally been recognized as benign, but there have been reports in single cases of cardiac-related deaths due to lethal arrhythmias and unknown dilated cardiomyopathy [7]. In a recently published paper, a subtle pre-clinical cardiovascular involvement in WD was described since adolescence, in terms of an abnormal blood pressure response after 3 min of upright position as well as lower ankle-brachial index compared to healthy counterparts [8].

Prospective trials evaluating the underlying cause of arrhythmias and heart failure in WD are still missing. The aim of this study was to comprehensively investigate the signs of cardiac involvement in WD.

## Methods

The cardiac manifestation WD study is a prospective clinical and imaging study. From 2016 to 2017, 61 patients suffering from WD were consecutively included in the study. The WD ambulatory clinic at University Hospital Dresden of Technische Universität Dresden, Germany, recruited 34 patients. The remaining 27 patients were recruited from five different neurology departments from all over Germany. The diagnosis of WD was reviewed according to the scoring system provided by the 8th International Meeting on WD and Menkes disease [9].

We selected 61 age- and sex-matched controls without a familial relationship to the patients from our study database. Controls were free from cardiac disease based on clinical examination, cardiac echocardiography, and cardiac magnetic resonance imaging (CMRI). This study was approved by the local ethics committee, and written informed consent was obtained from all participants and the study protocol conforms to the ethical guidelines of the 1975 Declaration of Helsinki.

### Evaluation of WD severity

The quantification of clinical symptoms was performed in consensus, using the 55-item Unified Wilson’s Disease Rating Scale (UWDRS) [10]. The UWDRS consists of a neurological, a psychiatric, and a hepatic subscale, each one differentiating in anamnestic information from the last two to four weeks and present clinical data. Based on the present clinical status, UWDRS subscores were calculated for different WD typical symptom complexes: dystonia (sum of UWDRS items 11A, 22, 23, 25A, and 26A; range 0–28), dysarthria (item 10; range 0–4), parkinsonism (items 11B, 12, 14–16, 20, 24, 25C, 26C; range 0–76), ataxia (items 25B, 26B; range 0–8), and tremor (items 12, 13, 18, 21; range 0–44).

### Laboratory examination

A list of all serum values is found in Table 2 of the supplemental data.

### 24 h-ECG

While wearing an ambulatory electrocardiograph (ECG, CardioMem® CM 3000, Getemed) for 24 h, patients continued their daily activities and the following parameters were measured from 24 h recordings: mean heart rate, maximal heart rate, minimal heart rate, count of supraventricular and ventricular premature contractions (PSVC, PVC), and disturbance of rhythm (arrhythmia, tachycardia, bradycardia). In our study, the time domain heart rate variability measurements were the mean of the standard deviations of all normal sinus RR intervals for all 5-min segments (SDNN index) and triangular index (TI).

### Echocardiography

Conventional echocardiographic studies using standard views and echo techniques, as recommended by the American Society of Echocardiography, were performed for all patients by experienced investigators using an iE33 Echocardiography System (Philips, NL) [11, 12]. From four-chamber and two-chamber views, left ventricular end-diastolic and end-systolic volumes and ejection fraction (LVEF) were measured by the modified Simpson disk method. Additionally, for measurement of the right ventricular (RV) function, we used the tricuspid annular plane systolic excursion *(*TAPSE), which is an excellent surrogate parameter for the RV systolic function [13]. Left ventricular (LV) diastolic function was assessed by Doppler analysis.

### Statistical analysis

SPSS 20.0 (SPSS, Inc., Chicago, IL, USA) was used for statistical analyses. The results are presented as mean and standard deviation, as median with lower and upper quartiles (25th and 75th percentiles), or as percentages. For group comparisons, independent samples t-test and Wilcoxon signed-rank test were used. Correlations were evaluated with the Pearson’s correlation coefficient. 24 h-ECG recording were not available from controls. For statistical comparison with healthy individuals we used the data from Bonnemeier et al [14].

## Results

The mean age for the WD patients and the control patients was 44.3 years (31 men, 30 women) and 45.7 years (31 men, 30 women), respectively. An overview of the demographic data and clinical characteristics of the 61 examined WD patients and controls is presented in Table 1. WD specific data, including duration of illness, phenotype, incidence of exacerbation, liver fibrosis grading, liver transplantation, and medical treatment, are displayed in Additional file 1: Table S1.

**Table 1:** Baseline clinical characteristics

Variable	Patients (*n* = 61)	Controls (*n* = 61)
Sex		
Male	31 (50.8%)	31 (50.8%)
Female	30 (49.2%)	30 (49.2%)
Age, years	44.3 (15.2)	45.7 (13.7)
Cardiovascular risk factors and symptoms		
Arterial Hypertension	9 (14.8%)	12 (19.7%)
Diabetes mellitus	1 (1.6%)	–
Hyperlipoproteinemia	4 (6.6%)	6 (9.8%)
Obesity	1 (1.6%)	2 (3.3%)
CHD	–	–
Symptoms of heart failure	–	–

Data are presented as mean (SD) or n (%) unless otherwise stated

CHD: Coronary heart disease

### Echocardiography

The echocardiography data are shown in Table 2. There were no differences between the groups with respect to LVEF (*p* = 0.382). Five of 61 patients had a reduced left ventricular ejection fraction (LVEF) < 50%. In patients with depressed LVEF, coronary heart disease was excluded by cardiac computed tomography angiography.

**Table 2:** Echocardiographic characteristics of patients and controls

	Patients	Controls	*p*-value
Left ventricle			
LVEF, %	63.78 (2.7)	63.2 (2.4)	0.382
LVEDD in mm	45.6 (5.9)	46.8 (6)	0.43
LVESD in mm	29.3 (5.1)	30.5 (5.9)	0.48
IVST, mm	9.3 (1.4)	10 (2.2)	0.17
PWT, mm	9.2 (1.3)	9.9 (1.9)	0.06
Aortic root, mm	30.4 (3.7)	30.4 (3.1)	0.96
E/A ratio	1.3 (0.7)	1.3 (0.4)	0.22
Diastolic dysfunction			
I°	9 (14.8%)	–	**0.001**
II°	–	–	
III°	–	–	
Right ventricle			
RVEDD in mm	26.4 (4.5)	27.2 (4.5)	0.45
RVSP, mmHg	22.3 (4.1)	24.1 (3.7)	0.13
TAPSE, mm	23.6 (4.1)	25.1 (3.6)	0.15

Data are presented as mean (SD), or n (%) unless otherwise stated

LVEDD left ventricular end-diastolic diameter, LVESD left ventricular end-systolic diameter, IVST interventricular septal thickness, PWT posterior wall thickness, LVEF left ventricular ejection fraction, RVEDD right ventricular end-diastolic diameter, RVSP right ventricular systolic pressure, TAPSE tricuspid annular plane systolic excursion

Systolic right ventricular (RV) function, assessed by tricuspid annular plane systolic excursion (TAPSE) (*p* = 0.15), didn’t significantly differ between both groups. Pulmonary hypertension was not detectable in any of the WD patients. However, nine of 61 patients had a diastolic dysfunction grade I, which is significantly more frequent than in controls (*p* = 0.001). Mild valve regurgitation was a common finding in patients with WD, however no statistical significance (*p* = 0.26) to controls was obvious. None of the patients showed valve stenosis.

### Laboratory examinations

Laboratory data are shown in the Additional file 1: Table S2.

### UWDRS and exacerbation

We could detect no correlation between UWDRS and LVEF or TAPSE. However, a moderate significant correlation between UWDRS and NT-Pro BNP was obvious (Additional file 1: Table S3).

Eighteen patients had an acute exacerbation within the disease process. Four of them had elevated troponin levels. None of the patients without an exacerbation showed elevated cardiac biomarkers. Patients with an exacerbation of WD in medical history had higher troponin levels compared to those without (11.3 ± 4.7 vs 4.6 ± 1.2, *p* < 0.001).

At the time of cardiac imaging, one of these patients suffered from an acute exacerbation with elevated cardiac troponin. After successful de-coppering troponin levels decreased significantly (from 46 ng/L to 16 ng/L).

### 24 h electrocardiographic recording

The majority of WD patients showed a sinus rhythm (56 patients, 91.8%). Three patients had an ectopic atrial rhythm; one patient had atrial flutter; and in one patient a high-grade atrioventricular block was detectable, which required permanent pacemaker implantation. Premature supraventricular and ventricular ectopic contractions were a common finding in WD patients (50 patients with PSVC, 30 patients with PVC). On average, 226.7 PSVC (range 0;3979) and 216.3 PVC (range 0;6727) were seen, but we could not detect any potentially fast malignant arrhythmias.

### Autonomic function

The autonomic function assessed by triangular index (TI) and SDNN-index (SDNNi) is age dependent as described by Bonnemeier et al. which we refer to concerning our normal values [14]. Therefore, Online Additional file 1: Figure S1 and S2 show a comparison of autonomic function parameters on the basis of age. With the exception of patients and controls in the 50–59 year old group, TI was significantly reduced in WD patients. In addition, SDNNi showed significantly lower values in the 40–49 and 50–59 years old age categories.

## Discussion

To date, this is the largest prospective clinical and imaging study evaluating a cardiac manifestation of patients suffering from WD. We found that signs of structural heart disease with reduced LVEF were obvious in 5 of the 61 patients.

Furthermore, patients who are severely affected by WD have higher NT-pro BNP levels and patients with an exacerbation in medical history have higher troponin than those without.

WD is an inherited autosomal recessive disease, characterized by an inability of the

liver to excrete copper in the bile, resulting in excessive copper deposition in the body, primarily in the liver and the brain [1]. Copper toxicity to the liver has been demonstrated in several models. The generally cited mechanism of pathology development in WD involves oxidative damage due to copper overload and intramitochondrial membrane crosslinking that culminates in mitochondrial destruction and liver failure [15, 16].

There is still no clear evidence of how and to what extent excessive copper accumulations leads to cardiac dysfunction. Cardiac involvement in WD was first described by Kuan [7] in 1987. He followed-up with a cohort of 53 WD patients. During that period, one patient died due to cardiac heart failure and another one due to repeated ventricular fibrillations. Several studies [5, 17, 18] have reported a broad spectrum of electrocardiographic findings without any clear, unified cardiac diagnosis. A mildly increased QRS in WD patients (92 ms versus 87.5 ms in controls), a prolongation of P-wave dispersion, and a higher risk for atrial fibrillation were reported. In our study, we used 24 h Holter monitoring to detect rhythm disturbances. The majority of our patients had supraventricular ectopic beats (84%) and about half of the WD patients had premature ventricular contractions, but the clinical relevance of these findings is difficult to evaluate. Premature ventricular contractions are common and have been described in 40–69% of healthy low-risk individuals as detected by 24 h ambulatory ECG recordings [19, 20]. Traditionally, PVC have been thought to be relatively benign in the absence of structural heart disease, but they also represent increased risk of ventricular dysfunction, sudden death, and all-cause mortality [21–23]. The presence of fibrotic tissue is considered to be the basis for ventricular arrhythmias, which can be also detected in WD patients [24]. In an autopsy study, Factor et al. [4] found evidence of interstitial and replacement fibrosis and focal inflammatory cell inflammation to a variable degree in all of the nine cases studied. This might explain why PVC burden in our cohort was increased compared to the aforementioned studies. Therefore, we conclude that premature ventricular contractions are a sign of copper toxicity to the heart.

Mildly increased heart rate in Wilson’s disease was found in several studies, but the underlying mechanisms remained unknown [25]. One possible explanation might be a decreased HRV, which was observed in our WD patients. Depressed HRV was confirmed to be a clear predictor of heart failure and over- all mortality in several studies [26, 27]. However, the clinical implication remains unclear. The underlying mechanism between decreased HRV in the cited studies and WD patients might be different. Autonomic dysfunction in heart failure patients is mainly due to small fiber dysfunction of the peripheral nervous system [28]. Wilson’s disease mainly involves the central nervous system rather than the peripheral nervous system [29]. The brain structures commonly involved in Wilson’s disease contain important autonomic centers. In a study by Chu et al., the sympathetic central conduction time was prolonged in the Wilson’s disease patients, which favors a central mechanism [30].

A recently published longitudinal cohort study revealed that patients suffering from WD exhibited a statistically significant 55% increased risk of heart failure (HF) [5]. Because the study design lacked cardiac imaging, the authors could only speculate about an underlying cause. Before this current study describing 61 patients, the literature regarding cardiac imaging of WD has been restricted to a total of 42 in adults. Hlubocka et al. [6] showed that patients with WD had a mildly increased thickness of the interventricular septum and the posterior wall in comparison to healthy subjects, but without a difference of LV mass.

It remains unclear how WD patients develop heart failure, as recently shown by Grandis et al. [5]

In our cohort 5 of the 61 patients had a mildly reduced LVEF. Whether this is due to copper toxicity is still a matter of speculation. The mechanism might be a result of multiple issues. A correlation of the LVEF to the severity of illness assessed by the UWDRS or WD exacerbation in medical history was not obvious. However, patients with an exacerbation in medical history had higher troponin than those without, which might point to a copper related toxicity and inflammation to the myocardium. This hypothesis is supported by the fact, that in one patient suffering from acute exacerbation adequate medical therapy and successful de-coppering was able to decrease elevated troponin levels and thus reduce the effect of copper-overload inflammation.

According to previous mentioned data of Grandis et al., patients with WD who developed HF had a mean age of 66 ± 18 years old. In comparison, the mean age of our cohort was 46 years, indicating that the detected structural changes may become clinically relevant much later.

### Limitations

Some limitations of our study should be acknowledged. The majority of our cohort, mostly stabilized symptomatic patients or asymptomatic subjects, are treated for WD. We hypothesize that this is the reason we only detected minor cardiac manifestations of WD. The cardiac involvement could be more obvious in an untreated cohort. Second, it would be favorable to perform cardiac magnetic resonance imaging in WD patients, especially in those patients with reduced systolic function. This way, subclinical minor changes, e.g. fibrosis or inflammatory response due to copper deposition, could be detected.

## Conclusion

As previous data and our study demonstrate, WD has a cardiac involvement. Although heart and vessels are not the main target of WD, being liver and brain those mainly involved, the detection of this unique population, potentially predisposed to cardiovascular accidents suggests to enhance strategies of primary prevention. Therefore, we suggest that patients with signs and symptoms of structural heart disease should undergo further cardiac examination in addition to the interdisciplinary treatment team of WD.

## Additional file

Additional file 1: **Table S1** Clinical characteristics of the WD patients. **Table S2** Laboratory characteristics of the studied WD patients. **Table S3** Association between UWDRS and clinical characteristics. **Figure S1.** Comparison of SDNN-Index of WD patients and controls. *** *P* < 0.001. **Figure S2.** Comparison of Triangular Index of WD patients and controls. *** *P* < 0.001, ** *P* = 0.005, * *P* = 0.05. (ZIP 45 kb)

## Abbreviations

CHD: Coronary heart disease
CMRI: Cardiac magnetic resonance imaging
ECG: Electrocardiography
EDD: End
EDD: End-diastolic diameter
EF: Ejection fraction
ESD: End-systolic diameter
HF: Heart failure
IVST: Interventricular septal thickness
LV: Left ventricle
PSVC: Premature supraventricular ventricular contractions
PVC: Premature ventricular contractions
PWT: Posterior wall thickness
RV: Right ventricle
SD: Standard deviation
SDNN: Standard deviation of the normal to normal intervals
TAPSE: Tricuspid annular systolic excursion
TI: Triangular index
UWDRS: Unified Wilson’s Disease Rating Scale
WD: Wilson’s disease
