# Supplementary material for: Cardiac and autonomic function in patients with Wilson’s disease
Source: Orphanet J Rare Dis. 2019 Jan 28;14:22. doi: 10.1186/s13023-019-1007-7 (PMC6348666; doi:10.1186/s13023-019-1007-7)
Supplement: Supplementary file 1 — Table S1 Clinical characteristics of the WD patients. Table S2 Laboratory characteristics of the studied WD patients. Table S3 Association between UWDRS and clinical characteristics. Figure S1. Comparison of SDNN-Index of WD patients and controls. *** P < 0.001. Figure S2. Comparison of Triangular Index of WD patients and controls. *** P < 0.001, ** P = 0.005, * P = 0.05. (ZIP 45 kb) [file 13023_2019_1007_MOESM1_ESM.zip › Additional file 1.docx]

**Additional File 1**

| **Table 1: Clinical characteristics of the Wilson’s disease patients** | |
| --- | --- |
| Variable | **Data (n=61)** |
| Duration of disease, years | 24.9 (14.7) |
| Phenotypic presentation at the time of diagnosis ^i^ | |
| Hepatic ^a^ | 27 (44.3%) |
| Neurologic or psychiatric with symptomatic liver disease | 7 (11.5%) |
| Neurologic or psychiatric with symptomatic liver disease | 18 (29.5%) |
| Asymptomatic ^b,c^ | 9 (14.8%) |
| Phenotypic presentation at the time of investigation ^i^ | |
| Hepatic ^a^ | 27 (44.3%) |
| Neurologic or psychiatric with symptomatic liver disease | 21 (34.4%) |
| Neurologic or psychiatric without symptomatic liver disease | 4 (6.6%) |
| Asymptomatic ^c^ | 9 (14.8%) |
| Patients with acute exacerbation within the disease process ^e^ | 18 (29.5%) |
| Patients after liver transplantation | 4 (6.6%) |
| Patients with pathological cranial MRI ^f^ | 32 (52.5%) |
| Liver fibrosis according to METAVIR score ^g^ |  |
| F0 | 27 (44.3%) |
| F1 | 7 (11.5%) |
| F2 | 4 (6.6%) |
| F3 | 8 (13.1%) |
| F4 | 15 (24.6%) |
| Therapy | |
| Penicillamine | 37 (60.7%) |
| Trientine | 13 (21.3%) |
| Zinc | 7 (11.5%) |
| No Wilson’s disease medication ^h^ | 4 (6.6%) |
| Data are presented as mean (SD), median (IQR), or n (%) unless otherwise stated.  ^a^ Symptomatic liver disease or asymptomatic elevation of liver enzymes with exclusion of neurological  manifestation  ^b^ Genetic testing within family screening  ^c^ No evidence of hepatic or neurologic symptoms  ^e^ Wilson’s disease dependent impairment of health condition which leads to hospitalization  ^f^ Evidence of characteristic pathological changes in the cranial MRI  ^g^ Liver fibrosis according to Metavir score using Data of transient Elastography (Fibroscan®)  ^h^ Patients after liver transplantation ^i^ modified by Ferenci | |

´

| **Table 2: Laboratory characteristics of the studied Wilson’s disease patients** | | | |
| --- | --- | --- | --- |
| Variable | Data (n=61) | Within normal range | Pathological values |
| Total CK, µmol/(s*l) | 1.7 (1.0) | 52 (85%) | 9 (15%) |
| CK-MB, µmol/(s*l) | 0.3 (0.1) | 55 (90%) | 6 (10%) |
| Myoglobin, µg/l | 30.9 (24.0) | 53 (87%) | 8 (13%) |
| Troponin T, ng/l | 4.0 (2.0) | 57 (93%) | 4 (7%) |
| NT-proBNP, ρmol/l | 8.6 (6.8) | 61 (100%) | - |
| Total serum copper, µmol/l | 6.4 (4.8) | 9 (15%) | 52 (85%) |
| Non-caeruloplasmin-bound serum copper, µmol/l | 2.9 (6.1) | 31 (51%) | 30 (49%) |
| Urinary copper excretion under medication, µmol/24h | 8.4 (9.2) | 30 (49%) | 31 (51%) |
| Urinary copper excretion without medication, µmol/24h | 1.8 (3.5) | 56 (91%) | 5 (9%) |
| Serum caeruloplasmin, g/l | 0.1 (0.1) | - | 61 (100%) |
| Serum iron, µmol/l | 18.8 (7.6) | 49 (80%) | 12 (20%) |
| Serum ferritin, µg/l | 178.8 (156.2) | 45 (74%) | 16 (26%) |
| ALT, µmol/(s*l) | 0.7 (0.5) | 42 (69%) | 19 (31%) |
| AST, µmol/(s*l) | 0.6 (0.3) | 40 (65%) | 21 (35%) |
| Total bilirubin, µmol/l | 9.4 (4.8) | 60 (98%) | 1 (2%) |
| γ-GTP, µmol/(s*l) | 0.8 (0.9) | 50 (82%) | 11 (18%) |
| ALP, µmol/(s*l) | 1.5 (0.6) | 57 (94%) | 4 (6%) |
| INR | 1.1 (0.1) | 54 (88%) | 7 (12%) |
| Albumin, g/l | 42.5 (3.8) | 60 (98%) | 1 (2%) |
| eGFR, ml/min/1,73 m^2^ | 81.9 (14.4) | 40 (66%) | 21 (34%) |
| Creatinine, µmol/l | 78.6 (24.6) | 53 (87%) | 8 (13%) |
| Data are presented as mean (SD), median (IQR), or n (%) unless otherwise stated.  ALT: alanine amino transferase; AST: aspartate amino transferase; γ-GT: gamma-glutamyl transpeptidase; ALP: alkaline phosphatase; INR: international normalized ratio; eGFR: estimated glomerular filtration rate according to Chronic Kidney Disease Epidemiology Collaboration; CK: creatine kinase | | | |

| **Table 3: Association between UWDRS and clinical characteristics** | | |
| --- | --- | --- |
|  | UWDRS |  |
| Variable | r | p value |
| LVEF | - 0.12 | 0.54 |
| TAPSE | - 0.18 | 0.39 |
| NT-pro BNP | 0.34 | **0.013** |

The correlation coefficient (r) was determined according to either Pearson or

Spearman depending on the data. LVEF: left ventricular ejection fraction;

RVEF: right ventricular ejection fraction, TAPSE: tricuspid annular plane systolic excursion

∗

P < 0.05.

**Additional Figure 1.** Comparison of SDNN-Index of WD patients and controls. *** P < 0.001.

**Additional Figure 2.** Comparison of Triangular Index of WD patients and controls. *** P < 0.001, ** P = 0.005, * P = 0.05.
