# Supplementary figures and images for: Cardiac and autonomic function in patients with Wilson’s disease
Source: Orphanet J Rare Dis. 2019 Jan 28;14:22. doi: 10.1186/s13023-019-1007-7 (PMC6348666; doi:10.1186/s13023-019-1007-7)

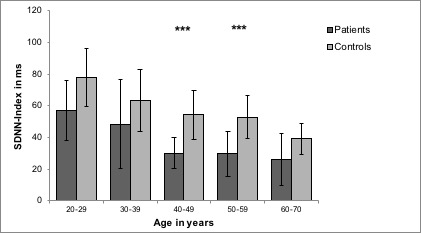

Supplement: Supplementary file 1 — Table S1 Clinical characteristics of the WD patients. Table S2 Laboratory characteristics of the studied WD patients. Table S3 Association between UWDRS and clinical characteristics. Figure S1. Comparison of SDNN-Index of WD patients and controls. *** P < 0.001. Figure S2. Comparison of Triangular Index of WD patients and controls. *** P < 0.001, ** P = 0.005, * P = 0.05. (ZIP 45 kb) [file 13023_2019_1007_MOESM1_ESM.zip › Additional figure 1.jpg]

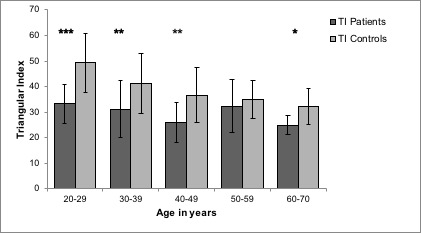

Supplement: Supplementary file 1 — Table S1 Clinical characteristics of the WD patients. Table S2 Laboratory characteristics of the studied WD patients. Table S3 Association between UWDRS and clinical characteristics. Figure S1. Comparison of SDNN-Index of WD patients and controls. *** P < 0.001. Figure S2. Comparison of Triangular Index of WD patients and controls. *** P < 0.001, ** P = 0.005, * P = 0.05. (ZIP 45 kb) [file 13023_2019_1007_MOESM1_ESM.zip › Additional figure 2.jpg]
